# Macrominerals and Trace Minerals in Commercial Infant Formulas Marketed in Brazil: Compliance With Established Minimum and Maximum Requirements, Label Statements, and Estimated Daily Intake

**DOI:** 10.3389/fnut.2022.857698

**Published:** 2022-04-28

**Authors:** Cristine Couto Almeida, Diego dos Santos Baião, Paloma de Almeida Rodrigues, Tatiana Dillenburg Saint'Pierre, Rachel Ann Hauser-Davis, Katia Christina Leandro, Vania Margaret Flosi Paschoalin, Marion Pereira da Costa, Carlos Adam Conte-Junior

**Affiliations:** ^1^Graduate Program in Sanitary Surveillance (PPGVS), National Institute of Health Quality Control (INCQS), Oswaldo Cruz Foundation (FIOCRUZ), Rio de Janeiro, Brazil; ^2^Center for Food Analysis (NAL), Technological Development Support Laboratory (LADETEC), Federal University of Rio de Janeiro (UFRJ), Rio de Janeiro, Brazil; ^3^Graduate Program in Veterinary Hygiene (PPGHV), Faculty of Veterinary Medicine, Fluminense Federal University (UFF), Niterói, Brazil; ^4^Laboratory of Advanced Analysis in Biochemistry and Molecular Biology (LAABBM), Department of Biochemistry, Federal University of Rio de Janeiro (UFRJ), Rio de Janeiro, Brazil; ^5^Departamento de Química, Pontifícia Universidade Católica do Rio de Janeiro (PUC-Rio), Rio de Janeiro, Brazil; ^6^Laboratory of Evaluation and Promotion of Environmental Health, Instituto Oswaldo Cruz, Oswaldo Cruz Foundation (FIOCRUZ), Rio de Janeiro, Brazil; ^7^Graduate Studies in Food Science (PPGCAL), Institute of Chemistry (IQ), Federal University of Rio de Janeiro (UFRJ), Rio de Janeiro, Brazil; ^8^Graduate Studies in Chemistry (PGQu), Institute of Chemistry (IQ), Federal University of Rio de Janeiro (UFRJ), Rio de Janeiro, Brazil; ^9^Laboratory of Inspection and Technology of Milk and Derivatives (LaITLácteos), School of Veterinary Medicine and Animal Science, Federal University of Bahia (UFBA), Salvador, Brazil

**Keywords:** supplementation, infant formula, macrominerals, ICP-MS, trace minerals

## Abstract

Infant formulas are the main nutritional source for infants when breastfeeding is not possible or recommended. The daily need for specific nutrients, such as essential minerals, in early stages of a child's life is high because of rapid infant growth and development, which impose metabolic flux increases on these pathways to support growth, physical activity, and defense against infections. In this context, this research aimed to determine macromineral and trace mineral contents in starting (phase 1) and follow-up (phase 2) infant formulas marketed in Brazil (*n* = 30) by inductively coupled plasma-mass spectrometry, calculate estimated daily intakes, and compare them to reference values regarding adequate intake and tolerable upper intake levels. The highest concentrations of macrominerals were observed in Ca, K, P, and Na, and trace minerals in Fe, Zn, Mn, and Cu. Certain homogeneity only to trace mineral contents was observed when analyzing inter-batch values from same manufacturers. In general, all phase 1 and phase 2 infant formula brands and batches met or exceeded Fe, Zn, Cu, Mo, and Se contents when compared to maximum limits established by *Codex Alimentarius*. In addition, Zn contents in eight phase 1 and in four phase 2 infant formulas were above the contents established by the tolerable upper intake level for children aged 0–6 and/or 7–12 months, respectively. These findings highlight the need to expand regular infant formula inspection concerning nutritional quality, as some composition aspects of these foods must be improved to follow international guidelines, since ideal requirements for infant formula composition, quality, and safety interfere in child development and adult health.

## Introduction

Breast milk is indisputably the ideal food for infants due to its specific nutritional characteristics and optimal nutrient balance, being always available at the ideal temperature, and protection from pathogen contamination, while also displaying numerous immunological and psychological advantages in reducing infant morbidity and mortality (1, 2). Because of these, the World Health Organization (WHO), the United Nations Children's Fund (UNICEF), and the Brazilian Ministry of Health (MS), as well as other entities, such as the European Society of Gastroenterology, Hepatology and Pediatric Nutrition (ESPGHAN), recommend exclusive breastfeeding up the 6th month of life and complementation after the introduction of food diversification for up to 2 years or more (3–6).

Breast milk, almost complete food and a primary nutrient source for infants and children, is composed of water, macronutrients such as fats, carbohydrates and proteins, and micronutrients, including vitamins and minerals (7). The main macrominerals and trace minerals in human breast milk are Na, K, Cl, Ca, Mg, and P, and Fe, Zn, Cu, Mn, I, F, Se, Cr, Mo, and Co, respectively. The need for essential trace minerals after childbirth is often greater than during the fetal period because of the high activity of metabolic pathways involved in growth, repair, and development, and in infection or hazardous physiopathological condition defense (3, 4). Macrominerals and trace minerals account for about 4% of total human body mass and are extremely important during infant feeding, since each mineral performs essential and specific physiological functions (6, 8), and appropriate intake of several macrominerals and trace minerals is crucial for child growth and development (9, 10).

Trace elements or microminerals, on the other hand, are available at lower concentrations and make up <0.01% of human body mass (11). These elements participate in several important reactions in the human body. Both Ca and P, for example, are required for skeletal development and bone mineralization during the fetal development and postnatal periods (9, 12–14), while Mg acts as a cofactor in enzymes responsible for several metabolic activities, accounting for innate and acquired immune responses, neuromuscular signaling and muscle contraction, protein and nucleic acid metabolism and bone growth and plays a role in maturation of lymphoid cells and tissues, as well as several enzyme activities (12, 14). Mn is an essential nutrient for skeletal structure, amino acid metabolism, and cholesterol and carbohydrates synthesis, and acts as a cofactor in several enzymes (15, 16). Although Mn deficiency contributes to one or more clinical symptoms, as this element is involved in different metabolic processes, its deficiency is not clearly associated with inadequate food intake in healthy children (17). Fe is part of the heme complex present in hemoglobin, myoglobin, cytochromes, and other heme proteins essential for hematopoiesis transport, storage, and oxygen use during childhood, and Fe imbalances are known to impair growth and cognitive development (8, 18). Zinc is necessary for gastrointestinal digestion, neurological development, structural enzyme maintenance and functions, genic expression modulation, growth, and immunity (11, 18). Although breast milk is the ideal food for infants, breastfeeding is not always possible or recommended. In this case consumption of infant formulas prepared in accordance with current *Codex Alimentarius* standards to complement or replace breast milk is the best alternative. Most commercialized infant formulas use cow milk as the main basic raw material, since it is easy to obtain and has a low cost compared to milk from other species (19–21). The concentrations of most macro and trace minerals are higher in cow milk than in breast milk, but availabilities are significantly lower in cow milk than in breast milk (8). Thus, it appears that the mineral concentrations of cow milk are not sufficient to fulfill infant nutritional needs. Therefore, when compared to human milk, infant formulas exhibit lower Fe availability, mainly because these formulas contain higher caseins, which bind cations, including Fe, through phosphoserine clusters. This strong binding maintains Fe soluble at the alkaline gut pH but prevents its release in a free form available for absorption by the duodenal mucosa (22, 23). Thus, to compensate for this low Fe bioavailability, high amounts of this element must be added to infant formulas. However, when in excess, Fe may saturate lactoferrin, decreasing the natural bacteriostatic effect of this protein and allowing for opportunistic pathogen proliferation, which may result in intestinal epithelium damage and microscopic bleeding in sufficient amounts to cause iron-deficiency anemia (24). Calcium, on the other hand, is better absorbed from breast milk because of high Ca:P ratio compared to cow milk. However, Ca from cow milk can form insoluble soaps in the human intestinal lumen, causing obstructions (25, 26). The high P concentrations in cow milk determine its preferential duodenal absorption, causing neonatal hypocalcemia in human infants, a pathological condition more common among infants fed with infant formulas (27, 28). As cow milk is not suitable for children under 1 year of age, infant formulas must undergo technological changes in their macro and micronutrient composition to make them safer and better meet infant metabolic activity requirements, avoiding nutrient limitations or excesses and considering applicable formulation compositions for each child development stage requirements (6, 29, 30).

Macro and trace mineral compositions and their potential toxicological risks due to excess of certain minerals in infant formulas have also been investigated (21, 31, 32) but to a lesser extent. In most published reports, macro and trace mineral levels of only a few elements were determined, and limited studies have focused on major and trace mineral determination. Furthermore, macro and trace mineral compositions in those studies were not evaluated in all available infant formula brands or were evaluated in brands marketed only in the country where a study was carried out.

Nutritional requirements to satisfy physiological functions and prevent pathological conditions caused by mineral deficiencies or excesses may negatively affect children when not attained, as in the case of Fe-deficiency anemia and/or malnutrition, which significantly compromises child development and growth (24). Considering that infant formulas may be the only food source for infants, these trace nutrients are of paramount importance, so their contents should be regularly monitored in these products. Furthermore, despite manufacturer attempts to mimic breast milk macro and trace mineral compositions, these compounds do not display the same availability as in breast milk and must, therefore, be corrected according to availability results. In this context, this study aimed to evaluate essential mineral contents in popular infant formula brands marketed in Brazil for children aged 0–6 months, termed starting or phase 1 formulas, and for children aged 6–12 months, defined as follow-up or phase 2 formulas, assessing compliance with levels proposed by the *Codex Alimentarius* (33) and Brazilian standards (34, 35) and agreement with label statements. In addition, we also assessed if the intake of these minerals is adequate by comparing it to dietary reference intakes (DRIs).

## Materials and Methods

### Sample Selection

The main brands available in different commercial establishments in the city of Rio de Janeiro, Brazil were selected. All products were registered by the Brazilian regulatory agency ANVISA and were packaged in cans, labeled according to manufacturer, and in powder form. Regarding composition, the following inclusion criteria were employed: (1) all infant formulas analyzed should be composed essentially of cow milk; (2) unhydrolyzed cow milk proteins; (3) with lactose as the main carbohydrate, and (4) supplemented with DHA and ARA for children aged 0–6 months (phase 1 starting formulas) and for children aged 6–12 months (phase 2 follow-up formulas). Infant formulas containing protein sources other than cow milk, such as soy or wheat protein, and infant formulas designed for specific needs, such as lactose-free or hydrolyzed, were not included in this study. Considering the products sold in the Brazilian market that meet the inclusion criteria, 10 formulations produced by three different manufacturers were evaluated. Thus, five phase 1 and five phase 2 formulas comprising three distinct batches of each brand were selected, totaling 30 samples (*N* = 30). Because of ethical reasons, manufacturers and brands are not disclosed, and samples are coded, where brands are represented by two capital letters, followed by the recommended phase (1 or 2), and batches of each brand are identified using capital letters (A, B, or C; Supplementary Table 1).

All the selected infant formulas are marketed in several commercial establishments, conveniently available to be purchased by consumers in the most populated part of the city of Rio de Janeiro. Although no formal inquiry was performed, as the selected formulas are commonly supplied in large supermarkets in town, they are considered as in high demand by consumers and were, thus, evaluated herein.

### Reagents

All reagents were of analytical grade. Ultra-pure water (resistivity > 18.2 MΩ cm) was obtained from a Milli-Q® INTEGRAL 10 system (Millipore, MA, United States). Nitric acid (Vetec, Rio de Janeiro, Brazil) was purified by sub-boiling followed by distillation in a quartz still (Kürner Analysentechnik, Rosenheim, Germany). The multi-element standard solution Merck IV (Merck, São Paulo, Brazil), comprising 29 elements in diluted nitric acid, was employed to prepare standard analytical curves. Two reference materials, certified Skimmed Milk Powder ERM®-BD150 (European Commission, Belgium) and Milk Powder 1549 (NIST SRM, United States) were used to assess method accuracy.

### Sample Preparation and Mineral Determination

Essential mineral concentrations were determined according to de Oliveira et al. (36), following the US EPA method 6020B employing inductively coupled plasma mass spectrometry (ICP-MS). Briefly, about 100 mg of each sample were transferred to sterile 15-ml screw-capped polypropylene tubes, and 1 ml of concentrated sub-boiled distilled nitric acid was added to the mixture and left overnight at room temperature. The following day, acid digestions were completed by heating the samples at 100°C for ~4 h. After cooling, the samples were adequately diluted with Milli-Q water and analyzed on a NexIon 300X spectrometer (PerkinElmer, MA, United States). Instrumental ICP-MS conditions are displayed in Supplementary Table 2.

The certified reference materials (CRMs) Skimmed Milk Powder ERM®-BD150 (EC, BEL) and Non-Fat Milk Powder 1549 (NIST SRM, United States) and a five-point calibration curve ranging from 100 to 1,000 mg·L^−1^ were used to assess linearity. The respective correlation coefficients are in the range 0.97–1. LOD (limit of detection) and LOQ (limit of quantification) were determined for all minerals according to the Brazilian National Institute of Metrology, Quality and Technology—INMETRO (37), as LOD = 3 SD blank/slope of the curve and LOQ = 10 SD blank/slope of the calibration curve. The total content of each element in the infant formula samples was calculated from three samples analyzed separately by multi-element external calibration, converted, and expressed as mg·100 g^−1^.

### Daily Intake Estimated From the Consumption of Infant Formulas

The daily intake of minerals depends on both concentration of the elements in food matrix and daily food consumption. Estimated daily intake (EDI) is a concept introduced to consider these factors. Therefore, we used the following equation to estimate whether the macromineral and trace mineral intakes of phase 1 and phase 2 infant formulas are adequate for infants from 0 to 6 months and 7 to 12 months old, respectively:


(1) $$\begin{array}{r} \text{EDI}={\text{C}}_{\text{m}}\times \;{\text{C}}_{\text{d}} \end{array}$$

where EDI represents the estimated daily intake of macrominerals and trace minerals, expressed as mg per day for all minerals; C_m_ is the average concentration of each element in the analyzed batches of infant formula determined by ICP-MS expressed as mg·100g^−1^ of formula powder; C_d_ is the recommended daily consumption amount stated on the label of each phase 1 and phase 2 infant formula (suggested amount to be consumed expressed in grams per day). The approximate consumption per day is described in Supplementary Table 3.

These estimated daily intakes were compared to the values of dietary reference intake (DRI) recommended by the Institute of Medicine (38). For infants 0–6 months old, all recommendations are in the form of adequate intakes (AIs) based on the composition of human milk from healthy mothers. For the second 6 months of life (ages 7–12 months), infants start a mixed diet of human milk and solid foods. In this case, there are no evidently different nutrient needs, except for some minerals, such as Fe and Zn, which have relatively higher requirements. An estimated average requirement (EAR) for Fe and Zn was derived for this age group. Tolerable upper intake level (UL) was calculated only for Fe, Zn, and Se, since an established UL value for the other minerals has still has not been determined (Supplementary Table 4). It is important to mention that this estimate does not consider the fact that powdered infant formulas must be reconstituted with drinking water (which also contains minerals) prior to consumption.

### Assessing Compliance

Compliance was verified by assessing infant formula labels to verify if the declared values, as well as the ICP-MS-determined values, were within the established criteria for macrominerals and trace minerals by both the Brazilian legislation (34, 35) and by the *Codex Alimentarius* (33), and whether the experimentally obtained values were in accordance with what was stated on the labels.

### Statistical Analyses

Significant differences in infant formula macromineral and trace mineral contents and their respective batches were assessed by two-way analysis of variance (ANOVA) with repeated measures. An additional *post-hoc* analysis (Bonferroni correction) was performed when a significant *F* was found. Results were considered significant when *p* < 0.05. Data were expressed as means ± standard deviations (SDs). All statistical analyses were carried out using the GraphPad Prism software version 5 for Windows® (GraphPad Software, CA, United States).

## Results

### Method Accuracy and Precision

Since infant formulas are similar to milk powder, the accuracy of the applied methodology was verified by analyzing Skimmed Milk Powder ERM®-BD150 and Non-Fat Milk Powder 1549 CRMs (results are shown in Table 1). The total concentrations of the elements determined in the CRMs (experimental value) were compared to the certified values. The recovery values are adequate for all elements (Ca, Cu, Fe, K, Mg, Mo, Na, P, Se, Zn, Mn, and I), as displayed in Table 1, ranging from 76 to 113%, showing in this way a good accuracy of the methodology used in this study (39, 40). The LOD and LOQ obtained for each determined micromineral are displayed in Supplementary Table 5.

**Table 1:** Element content in the certified reference materials, expressed as mg·kg^−1^ dry weigh, and the recovery (in %) calculated for each element.

**Minerals**	**Reference materials (mg·kg** ^**−1**^ **)**					
	**Skimmed Milk Powder BD150**			**Milk Powder 1549**		
	**Experimental**	**Reference**	**Recovery**	**Experimental**	**Reference**	**Recovery**
Ca	12,506 ± 428.2	13,900 ± 800	90%	12,277 ± 336	13,000 ± 500	94.4%
Cu	1.2 ± 0.5	1.08 ± 0.06	110%	0.72 ± 0.1	0.7 ± 0.1	103.2%
Fe	3.5 ± 0.3	4.6 ± 0.5	76%	1.87 ± 0.3	1.78 ± 0.1	105%
K	16,829 ± 344	17,000 ± 700	99%	17,335 ± 230	16,900 ± 300	102.6%
Mg	1,304 ± 26.4	1,260 ± 100	103.5%	1,305 ± 26.2	1,200 ± 30	108.7%
Mo	-	-	-	0.342 ± 0.06	0.34 ± 0.10	100.5%
Na	3,495.6 ± 206	4,180 ± 190	84%	4,149 ± 82.5	4,970 ± 100	83%
P	11,688 ± 556.01	11,000 ± 600	106%	11,429 ± 133	10,600 ± 200	108%
Se	0.186 ± 0.005	0.188 ± 0.014	99.1%	0.105 ± 0.5	0.11 ± 0.01	95.5%
Zn	50.7 ± 2.1	44.8 ± 2.01	113%	50.3 ± 2.7	46.1 ± 2.2	109%
Mn	0.3 ± 0.06	0.29 ± 0.018	90%	0.3 ± 0.01	0.26 ± 0.06	103%
I	1.69 ± 0.04	1.73 ± 0.14	97.5%	3.29 ± 0.03	3.38 ± 0.02	97.2%

### Content of Macrominerals and Trace Minerals in Infant Formulas

Average macromineral (Ca, Mg, Na, K, and P) and trace mineral (Fe, Zn, Cu, Cr, Mo, Se, I, Co, and Mn) contents in phase 1 and phase 2 formulas were highly variable for most minerals (Table 2). No significant difference for trace minerals Co and Mn (*p* < 0.01) among all the infant formulas was observed. The highest trace mineral concentrations were observed for Fe, followed by Zn, Mn, and Cu, while trace minerals detected at lower concentrations comprised Co, followed by Cr, Mo, I, and Se. The highest macrominerals concentrations were observed for Ca, followed by K, P, and Na. In addition, comparing the macrominerals in infant formulas, Mg presented the lowest concentration. In general, samples ME2 and NN2 contained the highest Ca, K, P, and Na concentrations, while NC1, DM1, and DA1 contained the lowest Ca, Na, K, P, and Mg concentrations. Furthermore, no significant difference for Mg concentrations was observed between samples NC1 and NC2, NN1 and NN2, and DA1 and DA2, and for K concentrations in samples DA1 and DA2. Also, no significant difference in P concentrations was observed between samples DA1 and DA2.

**Table 2:** Average macromineral and trace mineral concentrations in phase 1 and phase 2 infant formulas popularly marketed in Brazil.

**IFs**	**Essential minerals (mg**·**100g**^**−1**^**)**													
	**Macrominerals**					**Trace minerals**								
	**Ca**	**Mg**	**Na**	**K**	**P**	**Fe**	**Zn**	**Cu**	**Cr**	**Mo**	**Se**	**I**	**Co**	**Mn**
ME1	356.8 ± 62.4^d^	46.5 ± 2.4^d^	143.1 ± 13.2^e^	396.9 ± 43.6^c, d^	295.8 ± 25.5^c^	5.205 ± 0.213^d^	3.374 ± 0.983^a, b^	0.262 ± 0.040^c^	0.028 ± 0.004^b^	0.036 ± 0.008^a^	0.035 ± 0.006^b, c^	0.036 ± 0.007^d, e^	0.001 ± 0.001^a^	0.483 ± 0.333^a^
ME2	586.2 ± 9.7^a^	51.9 ± 4.2^c, d^	239.6 ± 7.3^b^	569.0 ± 14.5^a^	359.8 ± 31.2^a, b^	6.951 ± 0.111^b^	2.935 ± 0.647^c^	0.175 ± 0.046^d^	0.035 ± 0.009^a, b^	0.031 ± 0.001^a^	0.031 ± 0.009^b, c^	0.048 ± 0.005^c^	0.001 ± 0.001^a^	0.316 ± 0.014^a^
NC1	250.1 ± 33.8^e^	57.1 ± 3.8^a, b^	118.0 ± 23.1^e^	329.3 ± 26.2^d, e^	185.4 ± 11.4^f^	5.499 ± 0.190^d^	4.044 ± 0.017^a^	0.355 ± 0.009^a^	0.025 ± 0.004^b^	0.009 ± 0.001^b^	0.037 ± 0.014^b, c^	0.055 ± 0.006^b^	0.001 ± 0.001^a^	0.333 ± 0.007^a^
NC2	545.1 ± 12.5^b^	61.4 ± 1.0^a^	211.7 ± 5.1^c^	495.9 ± 35.3^b, c^	351.8 ± 7.1^b^	6.982 ± 0.413^b^	3.495 ± 0.159^b, c^	0.312 ± 0.011^b, c^	0.032 ± 0.007^a, b^	0.007 ± 0.003^b^	0.042 ± 0.011^b, c^	0.051 ± 0.005^b, c^	0.001 ± 0.001^a^	0.279 ± 0.018^a^
NN1	359.0 ± 36.8^d^	58.4 ± 1.3^b^	180.3 ± 11.6^d^	379.9 ± 31.3^c, d^	240.4 ± 8.1^e^	5.517 ± 0.995^c, d^	4.307 ± 0.351^a^	0.368 ± 0.040^a, b^	0.024 ± 0.003^b^	0.010 ± 0.001^b^	0.026 ± 0.011^c^	0.062 ± 0.005^a, b^	0.001 ± 0.001^a^	0.379 ± 0.015^a^
NN2	558.1 ± 25.5ª^, *b*^	59.4 ± 1.0^a, b^	271.9 ± 5.1^a^	500.9 ± 6.6^b^	380.9 ± 5.5^a^	6.751 ± 0.444^b^	3.704 ± 0.171^b^	0.315 ± 0.022^b, c^	0.031 ± 0.005^b^	0.013 ± 0.005^b^	0.026 ± 0.011^c^	0.068 ± 0.004^a^	0.001 ± 0.001^a^	0.333 ± 0.026^a^
DM1	383.3 ± 14.9^d^	39.0 ± 1.4^f^	141.9 ± 7.4^e^	331.7 ± 5.2^e^	256.4 ± 13.1^d, e^	6.204 ± 0.622^b, c^	2.942 ± 0.170^d^	0.311 ± 0.004^b, c^	0.024 ± 0.002^b^	0.024 ± 0.006^a^	0.040 ± 0.005^b, c^	0.038 ± 0.003^d^	0.001 ± 0.001^a^	0.308 ± 0.007^a^
DM2	501.6 ± 22.0^c^	43.1 ± 0.2^e^	183.6 ± 28.1^c, d^	427.6 ± 53.8^c^	346.8 ± 4.1^b^	8.310 ± 0.214^a^	4.264 ± 1.160^a, b^	0.215 ± 0.012^d^	0.042 ± 0.004^a^	0.029 ± 0.001^a^	0.099 ± 0.041^a^	0.048 ± 0.004^b, c^	0.001 ± 0.001^a^	0.287 ± 0.004^a^
DA1	405.4 ± 33.3^d^	32.4 ± 2.9^g^	147.0 ± 10.1^e^	364.2 ± 25.7^c, d^	240.9 ± 28.8^d, e^	6.058 ± 0.183^c^	3.072 ± 0.325^c, d^	0.271 ± 0.035^c^	0.023 ± 0.003^b^	0.011 ± 0.003^b^	0.033 ± 0.011^b, c^	0.040 ± 0.006^c, d^	0.001 ± 0.001^a^	0.325 ± 0.021^a^
DA2	487.7 ± 35.0^c^	31.6 ± 1.8^g^	178.0 ± 28.4^d^	417.2 ± 47.4^c^	251.0 ± 3.2^d, e^	7.604 ± 1.079^a, b^	3.787 ± 0.154^b^	0.257 ± 0.029^c^	0.030 ± 0.004^b^	0.025 ± 0.006^a^	0.040 ± 0.002^b^	0.028 ± 0.003^e^	0.001 ± 0.001^a^	0.286 ± 0.005^a^

*Data refer to three average batches from each brand of infant formulas marketed in Brazil. Each sample was analyzed in quintuplicate, and data are reported as means ± SD. Different superscript letters in the same column indicate significant differences among the IFs at a significance level of p <0.01. IFs, infant formulas*.

### Macromineral and Trace Mineral Contents in Batches of the Same Manufacturer

No inter-batches and brand differences were observed in almost all the trace elements, except for Fe, Zn, and Cu, where inter-batch and brand variations (Supplementary Table 6) were observed. Concerning Fe contents, inter-batch variations were observed in NN1, NC2, NN2, and DA2. Zn inter-batch variations were seen in ME1, DA1, ME2, DM2, and DA2. Furthermore, for Cu contents, inter-batch variations were observed in ME1, NN1, and ME2. No differences in macromineral concentrations among the three batches of the same manufacturer were noted for Ca in ME2 and NC2, Mg in DM2, Na in ME2 and NN2, K in DM1 and NN2, and P in NN2, DM2, and DA2.

### Macromineral and Trace Mineral Contents Detected and Declared on Infant Formula Labels

Compliance of detected macromineral and trace mineral concentrations in the infant formulas, corresponding declared label values, and the current legislation are displayed in Table 3. Brands with the highest number of inadequacies were NN1, DM1, NN2, DM2, and DA2, in which more than half of the evaluated minerals had values with a discrepancy of ±20% from the declared value on the labels. Concerning each mineral, Ca was discrepant in ME1, NC1, NN1, and NN2; Mg in ME1, DM1, DA1, DM2 and DA2; Na in DM1 and DM2; K in ME1, NC1, NN1, DM1, DA1, ME2, DM2, and DA2; P in NN1, NN2, and DA2; Fe in NN1 and NN2; Zn in NC1, NN1, DM1, DA1, ME2, NC2, NN2, and DA2; Cu in ME2, NN2, and DM2. Se and Mn were 20% above label values in all the infant formula brands, while I was 20% lower than label values in all the brands (except for DM2). Regarding the trace minerals Cr, Mo, and Co, Brazilian legislation (34, 35) and the *Codex Alimentarius* (33) have not yet defined minimum and maximum values for these elements, which explains why the contents are not expressed on the labels, making it impossible to assess conformity.

**Table 3:** Contents of macrominerals and trace elements expressed as mg·100 kcal^−1^ estimated in phase 1 and phase 2 infant formulas marketed in Brazil, contents are declared on their respective label and the minimum (Min.) and maximum (Max.) or guidance upper levels (GUL), as appropriate, recommended by the current legislation.

**Minerals**	**Phase 1 infant formulas (mg·100 kcal** ^**−1**^ **)**												
	**Standard values**			**ME1**		**NC1**		**NN1**		**DM1**		**DA1**	
	**Min**.	**Max**.	**GUL**	**Label**	**Estimated**	**Label**	**Estimated**	**Label**	**Estimated**	**Label**	**Estimated**	**Label**	**Estimated**
Ca	50	-	140	92	73.4*	63.4	49.5*	110.7	72.2*	79.1	75.5	94.0	83.8
Mg	5	-	15	12.1	9.6*	11.7	11.3	11.9	11.8	11.6	7.7*	12.2	6.7*
Na	20	60	-	30	29.4	25.7	23.4	33.2	36.3	37.4	27.9*	35.7	30.4
K	60	180	-	104	81.7*	95.0	65.2*	116.7	76.4*	96.9	65.3*	99.4	75.2*
P	25	-	100	53	60.9	33.7	36.7	62.4	48.4*	49.6	50.5	57.2	49.8
Fe	0.45	1.3	-	1	1.1	1.1	1.1	1.6	1.1*	1.2	1.2	1.3	1.3
Zn	0.5	-	1.5	0.720	0.700	1.100	0.792*	1.200	0.865*	0.800	0.579*	0.900	0.640*
Cu	0.035	-	0.120	0.063	0.062	0.079	0.079	0.072	0.080	0.063	0.061	0.068	0.056
Cr	NS	NS	NS	NS	0.006	NS	0.005	NS	0.005	NS	0.005	NS	0.005
Mo	NS	NS	NS	NS	0.007	NS	0.002	NS	0.002	NS	0.005	NS	0.002
Se	0.001	-	0.009	0.002	0.004**	0.003	0.006**	0.003	0.004**	0.002	0.008**	0.004	0.007**
I	0.010	-	0.060	0.016	0.007*	0.024	0.011*	0.024	0.012*	0.015	0.007*	0.022	0.008*
Co	NS	NS	NS	NS.	0.0002	NS	0.0002	NS	0.0002	NS	0.0002	NS	0.0002
Mn	0.001	-	0.100	0.013	0.103**	0.022	0.059**	0.022	0.080**	0.010	0.061**	0.018	0.062**
**Minerals**	**Phase 2 infant formulas (mg·100 kcal** ^**−1**^ **)**												
	**Standard values**			**ME2**		**NC2**		**NN2**		**DM2**		**DA2**	
	**Min**.	**Max**.	**GUL**	**Label**	**Estimated**	**Label**	**Estimated**	**Label**	**Estimated**	**Label**	**Estimated**	**Label**	**Estimated**
Ca	50	-	140	118	119.6	120	115	80.5	118.2**	114.7	103.9	120.1	106.7
Mg	5	-	15	12	10.6	n.d.	13	12.5	12.6	12.2	8.9*	12.9	6.9*
Na	20	60	-	45	48.9	44.3	44.7	50.8	57.6	49.7	38.0*	40.7	38.9
K	60	180	-	152	116.1*	126.6	104.6	132.4	106	133	88.5*	130.4	91.3*
P	25	-	100	64	73.4	69.6	74.2	44.5	80.7**	70.4	71.8	70	54.9*
Fe	0.9	2.0	-	1.6	1.4	1.6	1.5	1.2	1.4**	1.8	1.7	1.8	1.7
Zn	0.5	-	1.5	0.800	0.592*	1.100	0.738*	1.200	0.784*	1.000	0.882	1.100	0.832*
Cu	0.035	-	0.120	0.075	0.041*	0.076	0.063	0.085	0.064*	0.064	0.043*	0.067	0.057
Cr	NS	NS	NS	n.d.	0.007	n.d.	0.007	n.d.	0.007	n.d.	0.009	n.d.	0.007
Mo	NS	NS	NS	n.d.	0.006	n.d.	0.001	n.d.	0.003	n.d.	0.006	n.d.	0.005
Se	0.001	-	0.009	0.002	0.006**	0.002	0.007**	0.002	0.005**	0.003	0.020**	0.003	0.009**
I	0.010	-	0.060	0.020	0.010*	0.027	0.011*	0.032	0.014*	0.002	0.010**	0.019	0.006*
Co	NS	NS	NS	n.d.	0.0012	n.d.	0.0014	n.d.	0.0014	n.d.	0.0012	n.d.	0.0010
Mn	0.001	-	0.100	0.015	0.061**	0.010	0.063**	0.013	0.064**	0.011	0.060**	0.013	0.066**

All values were converted to milligrams and are expressed in 100 kcal (mg·100 kcal^−1^). The amount of carbohydrates and proteins expressed in grams was multiplied by 4 and the amount of lipids was multiplied by 9 to obtain calorie values (in kcal). After summing the total caloric values (corresponding 100 g of product), the macromineral and trace element results were calculated by 100 g of product and corrected to 100 kcal. Data followed by the (*) symbol indicate concentrations that are 20% below the content declared on the label, whereas the (**) symbol indicate concentrations above 20% the declared content. NS, not specified. Guidance upper levels (GULs) are for nutrients without sufficient information for a science-based risk assessment and are derived based on nutritional requirements of infants and an established history of apparently safe use. Minerals contents in infant formulas should not exceed GULs. Sources: CODEX, 2007 (33); BRASIL, 2011a (34); BRASIL, 2011b (35).

When the minimum and maximum or guidance upper level contents recommended by current legislations were compared with the detected macromineral and trace mineral concentrations (Table 3), only Se concentrations in DM2 (0.02 mg^.^kcal^−1^) and I in ME1 (0.007 mg^.^kcal^−1^), DM1 (0.007 mg^.^kcal^−1^), DA1 (0.008 mg^.^kcal^−1^), and DA2 (0.006 mg^.^kcal^−1^) were not compliant with recommendations.

### Individual Mineral Daily Intake

Mineral daily intakes considering the reference values of the Institute of Medicine (38) recommended for children aged 0–6 (phase 1 infant formulas) and for children aged 7–12 months (phase 2 infant formulas) are shown in Supplementary Table 7. Mineral contents found in the infant formulas (expressed in mg**·**100g^−1^) are shown in Supplementary Table 6 and are converted to mg**·**g^−1^. Then, the EDI of each mineral content was calculated by considering the daily consumption recommended by the manufacturers and included on the can labels (Supplementary Table 3). The estimated average intake of K (in the NC1A, NC1C, DM1A, DM1B, DM1C, and DA1B samples), Cu (in the ME1C sample), and I (in all the phase 1 infant formulas) calculated from the consumption of the investigated formulas was lower than recommendation values for AI (0–6 months). The average intake of Ca (282.9–514.7 mg**·**day^−1^), K (365.7–522.5 mg**·**day^−1^), and P (219.1–372.8 mg**·**day^−1^) was highest when compared with the other determined macrominerals of the phase 1 infant formulas. The highest average intake macrominerals observed in the phase 2 infant formulas were the same as that in the phase 1 infant formulas. Furthermore, the estimated average intake of Mg (in ME2B, ME2C, NN2A, NN2B, NN2C, DM2A, DM2B, DM2C, DA2A, DA2B, and DA2C), Na (in all the samples), K (in NC2A, NC2B, NN2A, NN2B, NN2C, DM2A, DM2B, DM2C, DA2A, DA2B, and DA2C), Cu (in ME2C), and I and Mn (in all the phase 2 infant formulas) was lower than recommendation values for AI/EAR (7–12 months).

**Figure 1:**
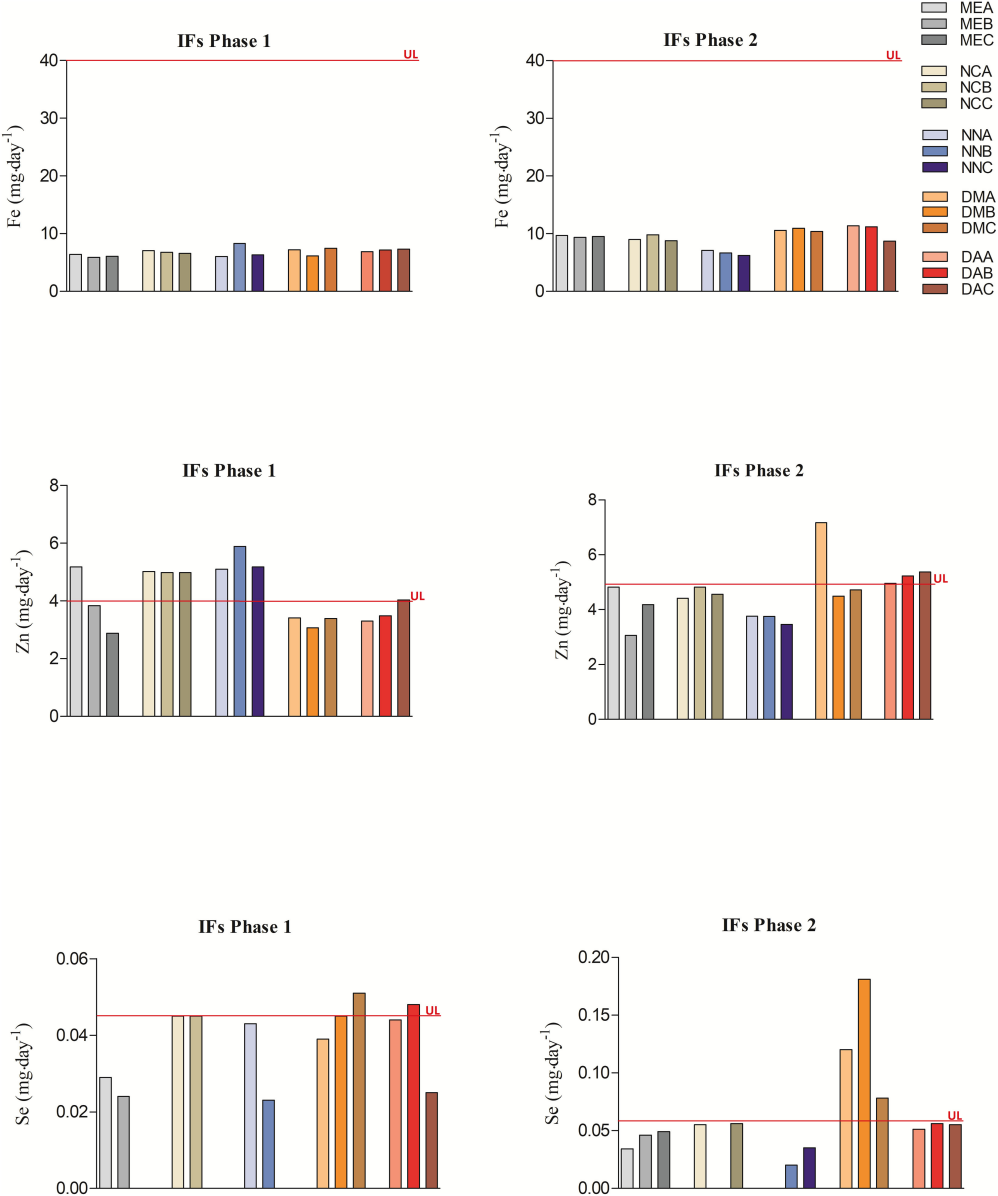
Daily intake values calculated for Fe, Zn, and Se of phase 1 and phase 2 infant formulas and their upper tolerable intakes for children aged from 0 to 12 months. Upper tolerable intake values according to current international legislation are shown in Supplementary Table 4. The daily intake values of Fe, Zn, and Se come from Supplementary Table 7. IFs, infant formulas; UL, upper tolerable level intakes.

Despite being essential at low concentrations, trace minerals, such as Fe, Zn, and Se, can be harmful when daily intakes exceed recommendations. Figure 1 displays the mean daily intake values calculated for Fe, Zn, and Se in all the formula brands and the UL recommended intakes for these elements for children aged from 0 to 12 months. Regarding Fe concentrations, neither the phase 1 nor the phase 2 infant formulas exceeded the tolerable limit of 40 mg**·**day^−1^. However, Zn concentrations in half of phase 1 and in four phase 2 infant formulas exceeded the tolerable limit of 4 mg·day^−1^ (0–6 months) and 5 mg·day^−1^ (7–12 months), respectively. The same results were noted for Se, where two phase 1 and three phase 2 infant formulas exceed the tolerable limit of 0.045 mg**·**day^−1^ (0–6 months) and 0.06 mg**·**day^−1^ (7–12 months).

## Discussion

Despite growing evidence on benefits of breastfeeding on health, psychosocial, and societal aspects, current breastfeeding rates worldwide are far from optimal (41). Health-compromised mothers using medication incompatible with breastfeeding or for reasons related to the baby's health, such as infants younger than 6 months old who are lactose-intolerant or display inadequate growth, breastfeeding cannot be performed. In this case, infant formulas formulated from cow milk or milk from other animals and mixed or not with other liquid ingredients, or reconstituted powders have proven suitable for human infant feeding (19–21). Furthermore, infant formulas have been frequently used recently, as mothers have been discouraged to breastfeed because of concerns regarding toxins found in the body that are often transferred to babies through lactation (42) or due to demands regarding work or school, life circumstances such as difficult home situations, and social problems with breastfeeding in public. However, even when there is no obstacle to breastfeeding, most infants are fed with some infant formula despite the strong endorsement of breastfeeding by the American Academy of Pediatrics (29). Despite manufacturer attempts to mimic breast milk nutritional compositions, these compounds may do not display the same availability as breast milk, and in this context, macromineral and trace mineral contents from the most popular infant formulas in Rio de Janeiro, southeastern Brazil, were evaluated herein.

Concerning the detected macrominerals, Ca contents were highest, followed by K, P, and Na. Regarding trace minerals, highest concentrations were observed for Fe, followed by Zn, Mn, and Cu. Macromineral and trace minerals contents were similar to those reported by Ikema et al. (43), of 385 ± 34.8, 344 ± 53.1, and 398 ± 71.1 μg·ml^−1^ Ca; 169 ± 17.1, 184 ± 44.6, and 192 ± 45.9 μg·ml^−1^ Na; 8.49 ± 1.21, 6.27 ± 2.36, and 9.3 ± 0.46 μg·ml^−1^ Fe; 3.49 ± 0.28, 3.21 ± 0.89, and 3.66 ± 0.79 μg·ml^−1^ Zn; 0.41 ± 0.06, 0.4 ± 0.14, and 0.49 ± 0.09 μg·ml^−1^ Cu; 0.06 ± 0.02, 0.068 ± 0.024, and 0.09 ± 0.04 μg·ml^−1^ Mn contents in milk-based powder infant formulas marketed in Nigeria, the United Kingdom, and the United States. The authors did not, however, evaluate K and P contents. In another assessment, Lesniewicz et al. (21) reported Ca (ranging from 279 to 703 mg·100g^−1^), P (ranging from 190 to 539 mg·100g^−1^), Fe (ranging from 3.5 to 7.4 mg·100g^−1^), Zn (ranging from 1.6 to 12 mg·100g^−1^) Cu (ranging from 0.068 to 0.502 mg·100g^−1^), and Mn (ranging from 0.026 to 0.374 mg·100g^−1^) as the most abundant macrominerals in milk-based powder infant formulas marketed in Poland and Spain. These authors did not, however, evaluate Na, K, Cu, and Mo contents. Furthermore, our results are similar to those reported by Moreno-Rojas et al. (7) who indicated Ca (478.9 ± 80.3 mg·100g^−1^), K (710.6 ± 137.4 mg·100g^−1^), P (294.1 ± 57.1 mg·100g^−1^), and Na (153.4 ± 43.6 mg·100g^−1^) as the most abundant macrominerals, and Fe (6.98 ± 3.04 mg·100g^−1^) and Zn (3.19 ± 0.86 mg·100g^−1^) as the most abundant trace minerals.

Furthermore, all macrominerals and trace minerals contents found in infant formulas were higher than those found in human milk, whereas those for Mn (0.317 mg·100g^−1^ and 0.0007 mg·100g^−1^, respectively) were even higher (Supplementary Table 4). The composition of milk from different species varies according to the biological needs of each species (44). Thus, for infants, human milk is more suitable than cow milk. Although cow milk and human milk contain a similar percentage of water, the relative amounts of carbohydrates, proteins, fats, vitamins, and minerals vary significantly between them. Regarding macrominerals, Ca content can be an excellent example of individual needs, comparing calves and human babies, since in cow milk, Ca content (107–133 mg·100g^−1^) reaches almost four times higher than in human milk (22–41 mg·100g^−1^), to fulfill the needs of calves that grow much faster and require more Ca to support growth (25, 26). However, the lower content of Ca in human milk is compensated by higher absorption rate when compared with cow milk. As stated by the Committee on Nutrition, the available data demonstrate that the bioavailability of Ca from human milk is greater than infant formula, increasing from 38%, as observed in formulas, to 58% in breast milk (45). Therefore, in an effort to solve this discrepancy, manufacturers add calcium with higher concentration in infant formulas to ensure Ca retention at least at comparable levels of Ca absorption (46). Although data on Ca toxicity in the 0- to 12-month age group are limited, it is known that high Ca intake in young children may increase the risk of Zn and Fe deficiencies, as Ca can reduce the absorption of these minerals (45). Other minerals, such as Na, K, and P, in cow milk have high renal solute loading when compared to human milk; excess unabsorbed solutes from diet have to be excreted in urine and may overload immature kidneys, forcing them to eliminate water from the body and, thus, increasing the risk of dehydration (47). In contrast to excess of some minerals, cow milk contains low concentrations of Fe, reinforcing why cow milk is considered unsuitable for children under 1 year of age (48, 49). In fact, a child younger than 6 months trying to reach the Fe recommended nutrient intake (EAR: 6.9 mg·day^−1^) would have to consume a significant extra amount of cow milk per day. Therefore, because of high and low concentrations of some minerals in cow milk, infant formula manufacturers try to adjust the concentrations to resemble breast milk and better meet child nutritional needs.

Ensuring homogeneity of nutrients present in the final product of different batches of the same brand is one of the fundamental characteristics for the nutritional quality of infant formulas. The nutritional content of a product is expected to maintain the same known nutritional standard, since, in many cases, these formulas are the only food offered to infants several times throughout the day, and exorbitant nutritional differences can lead to inappropriate child development (30, 32, 33). Certain homogeneity of trace mineral contents was observed when individually analyzing inter-batch values from the same manufacturers. Samples ME1C, NC1C, NN1C, NC2B, and NN2A were the only ones in which the mineral Se was not detected, with a value below the LOQ of 0.0015 mg·100g^−1^. However, macromineral contents varied among the batches of infant formulas from the same manufacturer for Ca in all the phase 1 infant formula brands and in NN2, DM2, and DA2; Mg among batches in all the phase 1 infant formula brands and in ME2, NC2, NN2, and DA2; Na among batches in all the phase 1 infant formulas brands and in NC2, DM2, and DA2; K among batches from brands ME1, NC1, NN1, DA1, NC2, DA1, ME2, NC2, DM2, and DA2; P among batches in all the phase 1 infant formulas brands and ME2 and NC2. An explanation for this difference may be the fact that the composition of milk, regardless of species, does not have exactly the same macro and micronutrient contents, as composition may vary according to different factors. In the case of infant formulas made from cow milk, variations in macromineral and trace mineral compositions may have been influenced by breed, lactation period, or diet of the animal (50), as well as by the processing conditions, purification method (membrane filtration vs. ion exchange), and thermal processing of infant formulas (50, 51).

Many companies that produce infant formulas add minerals to these products at concentrations higher than those present in breast milk to compensate for their lower availability, which in a way concerns the reliability of the nutrient values declared on the labels of these foods (33). The greatest differences observed in relation to the obtained and declared values on the labels were noted for Ca (except for DM1, DA1, ME2, NC2, NN2, DM2, and DA2), K (except for NC2 and NN2), Zn (except for ME1 and DM2), and I (except for DM2), with these being present at <20% of the declared value, while Se and Mn were present at a concentration 20% over the declared contents in all the brands of phase 1 and phase 2 infant formulas. Regarding comparison of the obtained results with standard values, it was noted that brand NC1 presented a Ca value (49.5 mg·100 kcal^−1^) lower than the recommended limit (50–140 mg·100 kcal^−1^). Furthermore, brands ME1, DM1, DA1, and DA2 presented an I value (0.007, 0.007, 0.008, and 0.006 mg·100 kcal^−1^) lower than the recommended limit (0.01–0.06 mg·100 kcal^−1^), respectively. The Se value detected in the DM2 brand (0.02 mg or 20 μg·100 kcal^−1^) was 222% above the guidance upper levels (GUL) of 0.009 mg·100 kcal^−1^. The excess Se observed in this sample reflects the value observed in the calculation of the daily intake, in which Se content was shown to be above the established tolerable limit. Furthermore, a higher value than the maximum tolerable intake level was noted when calculating the daily intake for Se and comparing it with the reference value (33–35). Considering a tolerance of 20% between the value declared on the labels and the ones experimentally obtained, in general, all brands contained only six minerals within the tolerance limits established by the Brazil, RDC, n° 360, from December 23, 2003 (52).

Furthermore, exposure to high Mn contents of infants who consumed infant formulas has recently been associated with adverse neurodevelopmental effects (53, 54). Considering Se contents, only the DM2 samples were above the maximum limit established in the legislation. The adverse effects caused by excess of Se are not yet well-documented. Evidence suggests that Se fortification at higher doses in infant formulas is safe, since it is estimated that Se intake of over 0.13, 0.16 mg·day^−1^ may trigger a toxic response in a 6-kg child. However, there is no experimental evidence of increased sensitivity to toxicity at younger ages. It is argued that inorganic forms of Se as sodium selenite can be more toxic than organic forms, selenomethionine, or Se-enriched yeast (55).

Some macrominerals and trace minerals do not have maximum recommendation values. In this case, GULs for nutrients without sufficient information for a science-based risk assessment are used. These levels are values derived on the basis of meeting nutritional requirements of infants and an established history of apparent safe use (33). They may be adjusted based on relevant scientific or technological progress. The purpose of GULs is to provide guidance to manufacturers, and they should not be interpreted as goal values (34). Nutrient contents in infant formulas should usually not exceed GULs unless higher nutrient levels cannot be avoided because of high or variable contents in constituents of infant formulas, or because of technological reasons. When a product type or form ordinarily contains levels lower than GULs, manufacturers should not increase levels of nutrients to approach GULs (33–35).

Infancy and early childhood are characterized by a very high growth rate, requiring a balanced diet rich in nutrients. Minerals and trace minerals are essential for biological processes, playing a vital role in normal growth and development (11). Minerals are involved in many important physiological functions, such as enzymatic reactions, bone mineralization, and cell and lipid protection in biological membranes, stimulating rapid growth and development. Low mineral bioavailability and intake may lead to deficiencies (21, 24, 31). Even if a child is breastfed for the first 6 months of life, complementary feeding is necessary after this period, and it is very important that these industrially produced foods, which are an important part of the diet of many young children, contain sufficient amounts if minerals, as infant feeding from birth up to the 1st years of life may influence an individual's entire future life (56). The World Health Organization (WHO) has made recommendations regarding daily mineral intake for infants and young children (19). The nutritional requirements for minerals should fulfill physiological functions and prevent deficiency symptoms (38). Dietary intake reference (DRI) values have been established to aid in dietary planning and to assess whether the intake of a particular nutrient meets the requirements of individuals or populations. When calculating the mineral intake resulting from infant formula consumption, in addition to the mineral concentration in infant formulas, the frequency of consumption of a child from 0 to 6 months or 7 to 12 months of age should also be considered. The result of this calculation is then compared to the EAR, AI, and/or UL used as dietary intake targets to be reached for healthy individuals (38). For children under 1 year of age, AI values are used instead of EAR values. However, in the case of Fe and Zn, for a child aged 7–12 months, the use of EARs is recommended, since this is a phase of greater demand for these minerals (57, 58).

This study verified whether consumption of infant formulas would meet or exceed the AI or EAR (Fe and Zn) for macrominerals and trace minerals analyzed. Comparing the EDI resulting from the consumption of the formulas with the reference values recommended by the Institute of Medicine (38), we identified that all the phase 1 infant formula brands and batches met or exceeded Ca, Mg, Na, P, Fe, Zn, Cu (except for ME1C), Cr, Mo, Se, and MN contents, and that all the phase 2 infant formula brands and batches met or exceeded Ca, P, Fe, Zn, Cu (except for ME2C), Cr, Mo, and Se contents when compared to the recommended values for the respective age groups. However, it was identified that all the brands and their phase 1 and 2 batches had I content below the recommended value for 0–6 months and for 7–12 months. Also, in all the phase 2 formula brands and batches, Na and Mn contents were below the 370 and 0.6 mg·day^−1^ recommendations (7–12 months), respectively. Furthermore, infant formulas NC1A, NC1C, DM1A, DM1B, DM1C, DA1B, NC2A, NC2B, NN2A, NN2B, NN2C, DM2A, DM2B, DM2C, DA2A, DA2B, and DA2C presented K contents below the AI recommendation (400 mg·day^−1^ for 0–6 months and 700 mg·day^−1^ for 7–12 months). Breast milk is capable of adequately nourishing children in the first 6 months of life; therefore, phase 1 infant formulas must be nutritionally similar to breast milk to meet the biochemical and physiological needs of a developing child (59). However, there is no such concern with phase 2 infant formulas, since from that period of 6 months onward, breastfeeding or intake of infant formula should be complemented with other foods to meet the nutritional needs of children and prevent malnutrition, nutritional deficiencies, and even childhood morbidity and mortality (60). Therefore, it should be considered that even if some trace element contents in phase 2 infant formulas are below the recommendation (AI or EAR), children in this age group are receiving macro- and micronutrients from other food sources that could supply infant needs.

As observed and mentioned above, the values of K and I essential trace minerals were found to be below the recommended values for most phase 1 infant formulas. The mineral I is essential in the metabolism of thyroid hormones (thyroxine and triiodothyronine) and helps regulate basal energy metabolism and reproduction. A deficiency of I can cause severe and irreversible mental retardation, deaf-muteness, congenital anomalies, as well as the most visible clinical manifestation, such as an enlarged thyroid gland, forming a goiter (61). Furthermore, K is the main intracellular cation. Its deficiency can influence the transmission of nerve impulses, which harms the maintenance of blood pressure and the control of skeletal muscle contraction (8). Therefore, considering that when breastfeeding cannot be performed and infant formulas are the only food source offered to infants aged 0–6 months, and that nutritional requirements of the diet during the infant's initial phase are highest, an inadequacy in the composition of macrominerals and trace minerals can negatively impact infant health.

On the other hand, some minerals can pose potential health hazards when consumed above the UL for an extended period of time. Excess of some minerals, such as Fe, Zn, and Se, can cause adverse health effects (10, 62, 63). To establish the maximum tolerable limit, UL, the highest value of daily intake of a nutrient that apparently does not result in adverse health effects, was used, supposing that if intake exceeds this limit, it should be considered a potential risk for adverse effects. Although the UL index has not yet been established for all macronutrients and trace minerals, or for the age groups considered herein, it is worth noting that the lack of UL does not mean that the risk of adverse effects does not exist from high mineral ingestion. The UL has been established for Fe, Zn, and Se (64), so the daily intakes for these elements were calculated. In this study, Fe estimated intake did not exceed the upper tolerable limit of 40 mg·day^−1^ in both the phase 1 and phase 2 formulas. However, Zn exceeded the established UL in phase 1 (in ME1A, NC1A, NC1B, NC1C, NN1A, NN1B, NN1C, and DA1C) and phase 2 (in DM2A, DA2A, DA2B, and DA2C) infant formulas of 4 and 5 mg·day^−1^, respectively. In addition, Se exceeded the established UL in phase 1 (in DM1C and DA1B) and phase 2 (in DM2A, DM2B, and DM2C) infant formulas of 0.045 and 0.06 mg·day^−1^, respectively. Long-term Zn intake over the optimal concentration can interfere in the metabolism of other trace minerals, such as Cu, reducing their absorption and causing anemia, in addition to affecting the immune system (65). In fact, decreased Cu uptake appears to be associated with high Zn intake (66, 67). The most common clinical features observed in humans concerning Se overexposure are hair loss, structural alterations of hair and nail keratin, and impaired skin development. In addition to these disorders, symptoms such as jaundice and gastrointestinal disorders were also observed (67, 68). However, it is important to note that none of these symptoms were observed or reported in early childhood.

Recommendations concerning infant formula composition refer to total nutrient contents as prepared ready for consumption according to the manufacturer's instructions. These generally inform consumers about the amount of powder and drinking water needed to reconstitute infant formula. The mineral intake by infants who consume infant formulas may be influenced by other factors not directly linked to actual product composition, as mineral concentrations in drinking water necessary to reconstitute the powder are not considered (69, 70). As a result, the concentrations of minerals in drinking water may significantly contribute to total macro and trace mineral infant intake. This is particularly applicable to formula-fed infants during the 1st months of life, which may be the most vulnerable group affected by excessive nutrient or contaminant concentrations in drinking water (71). The analytical results obtained in this study refer only to the analysis of infant formula prepared under standardized laboratory conditions with ultrapure water (resistivity > 18.2 MΩ cm), i.e., only the value determined directly on the powdered infant formulas. More representative studies on the actual mineral concentration in infants who consume infant formula would need to exclude or minimize the potential influences of different types of drinking water used in reconstitution. These studies would have to, however, be carried out using a standardized water supply or a ready-made liquid infant formula.

## Conclusion

In this study, the content of five macrominerals and nine trace minerals was determined in five phase 1 and five phase 2 powder infant formula samples from Brazil. Our findings indicate a certain homogeneity of trace mineral contents in inter-batch values from the same manufacturers. However, in some of the phase 1 and phase 2 infant formula samples, the content of Se was not detected because of values below the LOQ. The Ca, Mg, Na, K, and P contents varied among batches of the phase 1 and phase 2 infant formulas from the same manufacturer, and this could be attributed to variations in quality of raw materials, different manufacturing practices, finished products, and packaging containers.

Some macromineral and trace mineral contents presented values <20% of the declared value on labels, mainly in the phase 1 infant formulas. Furthermore, inadequacy regarding the maximum and minimum values established in the current legislation was only observed for I mainly in the phase 1 infant formulas.

Nutritional contents of all the phase 1 and phase 2 infant formula brands and batches were found to be lower than the AI or EAR values for the elements I in phase 1 and phase 2 infant formulas, and Na and Mn in the phase 2 infant formulas. In addition, elements K and Mg presented a value below the AI recommendation in almost all the phase 1 and phase 2 infant formula brands and batches. Infants fed with bottle formulations with low contents of essential elements may suffer from nutritional deficiencies and consequent health problems.

Despite numerous attempts by manufacturers, as well as specialists in the field of infant nutrition, to mimic breast milk composition and performance and the advances in technological processing experienced in the last years, which have made infant formulas safer and better for feeding children under the age of 1 year old, infant formulas still display marked differences in nutritional quality when compared to breast milk. Furthermore, the inter-batch differences detected herein indicate that the minerals in every batch are not strictly controlled. Although several public health bodies and agencies are responsible for developing standards for the formulation of infant foods, some aspects of their composition should be reevaluated and improved to follow current international legislation guidelines, as the ideal requirements for infant formula composition, quality, and safety must be followed carefully because of the major impact on adulthood population health linked to poor childhood nutrition or obesity.

## Data Availability Statement

The original contributions presented in the study are included in the article/Supplementary Material, further inquiries can be directed to the corresponding author.

## Ethics Statement

Written informed consent was not obtained from the minor(s)' legal guardian/next of kin for the publication of any potentially identifiable images or data included in this article.

## Author Contributions

CCA and PR carried out the experiments, interpreted the data, investigated previous studies for discussion, and drafted the manuscript with support from DB and RH-D. TS, MC, KL, VP, and CC-J supervised the project and critically reviewed and corrected the manuscript. All authors contributed to the article and approved the submitted version.

## Funding

The authors are thankful for the financial support provided by Fundação de Amparo à Pesquisa do Estado do Rio de Janeiro (FAPERJ) Brazil, process numbers [E-26/203.049/2017 and E-26/200.891/2021] and FAPERJ, PDR-10, process number [E-26/202.254/2018], Conselho Nacional de Desenvolvimento Científico e Tecnológico (CNPq), process number [311422/2016-0], and Coordenação de Aperfeiçoamento de Pessoal de Nível Superior (CAPES) Brazil, finance code 001.

## Conflict of Interest

The authors declare that the research was conducted in the absence of any commercial or financial relationships that could be construed as a potential conflict of interest.

## Publisher's Note

All claims expressed in this article are solely those of the authors and do not necessarily represent those of their affiliated organizations, or those of the publisher, the editors and the reviewers. Any product that may be evaluated in this article, or claim that may be made by its manufacturer, is not guaranteed or endorsed by the publisher.
