# Supplementary material for: Macrominerals and Trace Minerals in Commercial Infant Formulas Marketed in Brazil: Compliance With Established Minimum and Maximum Requirements, Label Statements, and Estimated Daily Intake
Source: Front Nutr. 2022 Apr 28;9:857698. doi: 10.3389/fnut.2022.857698 (PMC9096439; doi:10.3389/fnut.2022.857698)
Supplement: Supplementary file 1 [file Data_Sheet_1.PDF]

Supplementary file S1

Table S1. Codes for the commercial infant formulas evaluated in the present study.

Phase 1 code (0-6 months)	Batch code	Phase 2 code (6-12 months)	Batch code
**ME1**	ME1A<br>ME1B<br>ME1C	**ME2**	ME2A<br>ME2B<br>ME2C
**NC1**	NC1A<br>NC1B<br>NC1C	**NC2**	NC2A<br>NC2B<br>NC2C
**NN1**	NN1A<br>NN1B<br>NN1C	**NN2**	NN2A<br>NN2B<br>NN2C
**DM1**	DM1A<br>DM1B<br>DM1C	**DM2**	DM2A<br>DM2B<br>DM2C
**DA1**	DA1A<br>DA1B<br>DA1C	**DA2**	DA2A<br>DA2B<br>DA2C

Five phase 1 and five phase 2 formulas were selected, comprising three distinct batches of each brand totaling thirty samples (N = 30). Samples were coded, where brands are represented by two capital letters, followed by the recommended phase (1 or 2) and batches of each brand are identified using capital letters (A, B or C).
