# Supplementary material for: Macrominerals and Trace Minerals in Commercial Infant Formulas Marketed in Brazil: Compliance With Established Minimum and Maximum Requirements, Label Statements, and Estimated Daily Intake
Source: Front Nutr. 2022 Apr 28;9:857698. doi: 10.3389/fnut.2022.857698 (PMC9096439; doi:10.3389/fnut.2022.857698)
Supplement: Supplementary file 2 [file Data_Sheet_2.PDF]

Supplementary file S2

Table S2. ICP-MS conditions applied for mineral determinations.

**ICP-MS conditions**	**Characteristics**
Radiofrequency power	1100 W
Plasma flow	17.0 $L \cdot min^{-1}$
Auxiliary gas flow	1.2 $L \cdot min^{-1}$
Carrier gas flow	0.98 $L \cdot min^{-1}$
Skimmer composition	Pt
Dwell time	50 ms per isotope
Scanning mode	Peak hopping
Resolution	0.7 uma (u)
Scans by reading	5
