# Supplementary material for: Macrominerals and Trace Minerals in Commercial Infant Formulas Marketed in Brazil: Compliance With Established Minimum and Maximum Requirements, Label Statements, and Estimated Daily Intake
Source: Front Nutr. 2022 Apr 28;9:857698. doi: 10.3389/fnut.2022.857698 (PMC9096439; doi:10.3389/fnut.2022.857698)
Supplement: Supplementary file 3 [file Data_Sheet_3.PDF]

Supplementary file S3

Table S3. Recommended daily consumption amount stated on the labels of each phase 1 and phase 2 infant formula evaluated herein.

Approximate consumption per day (g)					
**Infant Formulas**	**ME1**	**NC1**	**NN1**	**DM1**	**DA1**
Phase 1 (0-6 months)	117.5	123.5	125.1	111.9	117.5
Phase 2 (7-12 months)	**ME2**	**NC2**	**NN2**	**DM2**	**DA2**
	137.0	131.6	98.7	128.0	137.0

Suggested average amount in grams per day.
