# Supplementary material for: Macrominerals and Trace Minerals in Commercial Infant Formulas Marketed in Brazil: Compliance With Established Minimum and Maximum Requirements, Label Statements, and Estimated Daily Intake
Source: Front Nutr. 2022 Apr 28;9:857698. doi: 10.3389/fnut.2022.857698 (PMC9096439; doi:10.3389/fnut.2022.857698)
Supplement: Supplementary file 4 [file Data_Sheet_4.PDF]

Supplementary file S4

Table S4. Recommended consumption values according to current international legislation, expressed as UL (tolerable upper intake level), EAR (estimated average requirement), AI (adequate intake), the ranges concentration of macrominerals and trace minerals in human milk and cow milk.

**Macrominerais**	***Dietary Reference Intakes (mg·day^{-1})***						***Human's Milk***	***Cow's Milk***
	**0-6 months**			**7-12 months**				
	**EAR**	**AI**	**UL**	**EAR**	**AI**	**UL**	***mg·100g^{-1}***	***mg·100g^{-1}***
Na	ND	110	ND	ND	370	ND	12-15	40-58
K	ND	400	ND	ND	700	ND	46-55	144-178
Ca	ND	210	ND	ND	260	ND	22-41	107-133
Mg	ND	30	ND*	ND	75	ND*	3.0-3.4	9-16
P	ND	100	ND	ND	275	ND	12-17	63-102
Fe	ND	0.27	40	6.9	11	40	0.02-0.06	0.02-0.03
Zn	ND	2	4	2.5	3	5	0.038	0.074-0.145
Cu	ND	0.2	ND	ND	0.22	ND	0.022-0.077	0.002-0.03
Mn	ND	0.003	ND	ND	0.6	ND	0.0007	0.0013-0.004
I	ND	0.11	ND	ND	0.13	ND	0.0005-0.009	0.002-0.006
Se	ND	0.015	0.045	ND	0.02	0.06	0.001-0.005	0.001-0.002
Cr	ND	0.0002	ND	ND	0.005	ND	0.0041	0.001-0.004
Mo	ND	0.002	ND	ND	0.003	ND	0.001	0.0024-0.006
Co	ND	ND	ND	ND	ND	ND	0.000114	0.00005-0.00013

AI, adequate intake; EAR, estimated average requirement; UL, tolerable upper intake level. ND, not determined; T, trace. The EAR or AI for Na and Co have not yet been established. Sources (38, 44).
