# Supplementary material for: Macrominerals and Trace Minerals in Commercial Infant Formulas Marketed in Brazil: Compliance With Established Minimum and Maximum Requirements, Label Statements, and Estimated Daily Intake
Source: Front Nutr. 2022 Apr 28;9:857698. doi: 10.3389/fnut.2022.857698 (PMC9096439; doi:10.3389/fnut.2022.857698)
Supplement: Supplementary file 5 [file Data_Sheet_5.PDF]

Supplementary file S5

Table S5. LOD and LOQ for each element determined in IF samples.

Minerals	LOD (mg·100g^{-1})	LOQ (mg·100g^{-1})
Ca	0.2818	0.9394
Mg	0.0010	0.0033
Na	0.0046	0.0155
K	0.0152	0.0506
P	0.0780	0.2601
Fe	0.0015	0.0015
Zn	0.0030	0.0101
Cu	0.0006	0.0021
Cr	0.0005	0.0017
Mo	0.0001	0.0002
Se	0.0045	0.0151
I	0.0006	0.0020
Co	0.0001	0.0004
Mn	0.0001	0.0004

IFs, infant formulas; LOD, limits of detection; LOQ, limits of quantification.
