# Supplementary material for: Macrominerals and Trace Minerals in Commercial Infant Formulas Marketed in Brazil: Compliance With Established Minimum and Maximum Requirements, Label Statements, and Estimated Daily Intake
Source: Front Nutr. 2022 Apr 28;9:857698. doi: 10.3389/fnut.2022.857698 (PMC9096439; doi:10.3389/fnut.2022.857698)
Supplement: Supplementary file 6 [file Data_Sheet_6.PDF]

*Supplementary file S6*

**Table S6.** Average macromineral and trace mineral contents in the three phase 1 and phase 2 infant formulas batches evaluated herein.

| IFs | Essential Minerals (mg·100 g$^{-1}$) | | | | | | | | | | | | | |
|---|---|---|---|---|---|---|---|---|---|---|---|---|---|---|
| | *Macrominerals* | | | | | *Trace minerals* | | | | | | | | |
| | **Ca** | **Mg** | **Na** | **K** | **P** | **Fe** | **Zn** | **Cu** | **Cr** | **Mo** | **Se** | **I** | **Co** | **Mn** |
| | ***Phase 1 infant formulas*** | | | | | | | | | | | | | |
| **ME1A** | 288.0±20.4[c] | 47.4±2.4[a] | 129.5±9.0[c] | 444.8±30.0[a] | 302.5±9.1[a] | 5.429±0.198[a] | 4.407±0.142[a] | 0.469±0.147[a] | 0.026±0.007[a] | 0.027±0.005[a] | 0.025±0.006[a] | 0.042±0.004[a] | 0.001±0.001[a] | 0.267±0.051[a] |
| **ME1B** | 409.8±18.6[a] | 48.4±0.1[a] | 143.8±3.1[b] | 386.6±5.8[b] | 317.4±27.8[a] | 5.006±0.217[a] | 3.266±1.083[a,b] | 0.172±0.074[b] | 0.033±0.001[a] | 0.042±0.010[a] | 0.021±0.008[a] | 0.037±0.004[a] | 0.001±0.001[a] | 0.866±0.694[a] |
| **ME1C** | 372.6±2.3[b] | 43.8±1.3[b] | 155.9±2.4[a] | 359.4±12.6[c] | 267.7±5.5[b] | 5.180±0.267[a] | 2.449±0.225[b] | 0.144±0.004[b] | 0.026±0.006[a] | 0.038±0.002[a] | n.d. | 0.028±0.014[a] | 0.001±0.001[a] | 0.315±0.006[a] |
| **NC1A** | 229.1±9.5[b] | 55.9±0.4[b] | 101.4±0.6[c] | 309.3±5.6[b] | 180.3±1.1[b] | 5.705±0.540[a] | 4.064±0.080[a] | 0.365±0.004[a] | 0.027±0.001[a] | 0.009±0.001[a] | 0.037±0.004[a] | 0.060±0.005[a] | 0.001±0.001[a] | 0.333±0.005[a] |
| **NC1B** | 289.0±8.5[a] | 61.4±0.5[a] | 144.4±12.8[a] | 359.4±12.8[a] | 198.4±1.5[a] | 5.463±0.338[a] | 4.033±0.224[a] | 0.350±0.015[a] | 0.027±0.002[a] | 0.008±0.001[a] | 0.037±0.004[a] | 0.049±0.009[a] | 0.001±0.001[a] | 0.326±0.0013[a] |
| **NC1C** | 232.1±29.2[b] | 54.7±3.6[b] | 108.1±0.6[b] | 319.7±32.8[a,b] | 177.4±2.9[b] | 5.329±0.335[a] | 4.036±0.146[a] | 0.349±0.024[a] | 0.021±0.006[a] | 0.010±0.001[a] | n.d. | 0.056±0.001[a] | 0.001±0.001[a] | 0.341±0.019[a] |
| **NN1A** | 316.8±15.4[b] | 59.1±0.5[a] | 167.7±3.7[b] | 344.3±15.8[b] | 238.3±1.6[b] | 4.838±0.449[b] | 4.073±0.128[a] | 0.348±0.011[b] | 0.026±0.001[a] | 0.009±0.001[a] | 0.034±0.006[a] | 0.063±0.001[a] | 0.001±0.001[a] | 0.362±0.026[a] |
| **NN1B** | 384.5±34.7[a] | 59.2±0.3[a] | 190.5±10.7[a] | 403.4±23.8[a] | 233.6±3.1[b] | 6.660±0.269[a] | 4.711±0.053[a] | 0.414±0.002[a] | 0.023±0.004[a] | 0.011±0.001[a] | 0.020±0.009[a] | 0.060±0.003[a] | 0.001±0.001[a] | 0.389±0.026[a] |
| **NN1C** | 375.6±1.7[a] | 56.9±0.3[b] | 182.7±0.7[a] | 392.1±6.8[a] | 249.4±11.7[a] | 5.054±0.162[b] | 4.138±0.123[a] | 0.342±0.008[b] | 0.024±0.001[a] | 0.011±0.001[a] | n.d. | 0.063±0.001[a] | 0.001±0.001[a] | 0.386±0.022[a] |
| **DM1A** | 366.1±6.9[b] | 37.7±0.1[c] | 148.1±1.3[a] | 331.0±9.4[a] | 241.4±0.1[c] | 6.450±0.060[a] | 3.049±0.172[a] | 0.312±0.002[a] | 0.024±0.002[a] | 0.024±0.001[a] | 0.035±0.013[a] | 0.040±0.001[a] | 0.001±0.001[a] | 0.316±0.005[a] |
| **DM1B** | 392.1±8.8[a] | 38.7±0.4[b] | 133.7±3.6[b] | 326.8±2.8[a] | 262.2±0.2[b] | 5.496±1.418[a] | 2.749±0.479[a] | 0.314±0.005[a] | 0.026±0.004[a] | 0.024±0.001[a] | 0.041±0.001[a] | 0.037±0.004[a] | 0.001±0.001[a] | 0.306±0.014[a] |
| **DM1C** | 391.5±21.9[a] | 40.5±0.3[a] | 143.9±19.8[a,b] | 337.2±31.0[a] | 265.5±2.2[a] | 6.666±0.813[a] | 3.031±0.061[a] | 0.306±0.030[a] | 0.021±0.001[a] | 0.025±0.001[a] | 0.045±0.004[a] | 0.035±0.003[a] | 0.001±0.001[a] | 0.301±0.015[a] |
| **DA1A** | 406.7±18.9[b] | 30.5±0.5[b] | 153.1±2.4[a] | 391.0±12.3[a] | 230.5±7.6[b] | 5.856±0.282[a] | 2.813±0.327[b] | 0.237±0.018[a] | 0.026±0.001[a] | 0.011±0.001[a] | 0.037±0.011[a] | 0.044±0.002[a] | 0.001±0.001[a] | 0.322±0.006[a] |
| **DA1B** | 371.6±8.7[c] | 30.9±0.1[b] | 135.4±2.9[b] | 339.7±9.3[c] | 218.7±5.6[b] | 6.105±0.561[a] | 2.967±0.017[b] | 0.267±0.008[a] | 0.022±0.003[a] | 0.012±0.001[a] | 0.041±0.001[a] | 0.040±0.002[a] | 0.001±0.001[a] | 0.306±0.024[a] |
| **DA1C** | 438.1±3.4[a] | 35.7±0.3[a] | 152.7±4.1[a] | 361.8±10.9[b] | 273.4±1.8[a] | 6.214±0.615[a] | 3.437±0.191[a] | 0.308±0.005[a] | 0.020±0.006[a] | 0.010±0.001[a] | 0.021±0.013[a] | 0.036±0.008[a] | 0.001±0.001[a] | 0.347±0.032[a] |
| | ***Phase 2 infant formulas*** | | | | | | | | | | | | | |
| **ME2A** | 596.9±29.3[a] | 58.1±1.7[a] | 234.5±2.2[a] | 573.3±1.1[a] | 394.9±26.1[a] | 7.063±0.105[a] | 3.516±0.484[a] | 0.225±0.050[a] | 0.035±0.001[a] | 0.031±0.001[a] | 0.025±0.009[a] | 0.043±0.008[a] | 0.001±0.001[a] | 0.326±0.011[a] |
| **ME2B** | 577.8±4.1[a] | 49.2±0.1[b] | 247.9±13.4[a] | 580.9±10.6[a] | 335.3±13.0[b] | 6.842±0.372[a] | 2.238±0.075[b] | 0.166±0.014[b] | 0.037±0.003[a] | 0.032±0.001[a] | 0.033±0.007[a] | 0.048±0.002[a] | 0.001±0.001[a] | 0.321±0.005[a] |
| **ME2C** | 584.0±4.7[a] | 48.8±3.0[b] | 236.3±3.9[a] | 552.9±4.0[b] | 349.3±6.8[b] | 6.948±0.091[a] | 3.052±0.149[a] | 0.134±0.024[b] | 0.034±0.004[a] | 0.031±0.001[a] | 0.036±0.006[a] | 0.052±0.012[a] | 0.001±0.001[a] | 0.300±0.027[a] |
| **NC2A** | 532.1±16.4[a] | 60.4±0.6[b] | 213.5±0.9[a] | 468.2±0.7[c] | 344.5±7.3[b] | 6.820±0.184[b] | 3.354±0.181[a] | 0.307±0.021[a] | 0.032±0.002[a] | 0.005±0.004[a] | 0.042±0.010[a] | 0.047±0.005[a] | 0.001±0.001[a] | 0.264±0.026[a] |
| **NC2B** | 546.3±16.6[a] | 61.4±1.1[a,b] | 205.9±3.9[b] | 484.1±3.1[b] | 352.3±7.4[a,b] | 7.451±0.373[a] | 3.667±0.100[a] | 0.324±0.001[a] | 0.032±0.001[a] | 0.010±0.003[a] | n.d. | 0.056±0.005[a] | 0.001±0.001[a] | 0.300±0.015[a] |
| **NC2C** | 557.0±7.7[a] | 62.5±0.6[a] | 215.7±9.6[a,b] | 535.6±43.3[a] | 358.6±5.6[a] | 6.675±0.572[a,b] | 3.463±0.070[a] | 0.305±0.028[a] | 0.032±0.001[a] | 0.005±0.003[a] | 0.042±0.001[a] | 0.049±0.004[a] | 0.001±0.001[a] | 0.274±0.011[a] |
| **NN2A** | 563.4±13.4[a] | 59.3±3.2[a,b] | 266.5±1.9[a] | 501.2±1.3[a] | 385.4±32.2[a] | 7.202±0.056[a] | 3.805±0.189[a] | 0.334±0.017[a] | 0.031±0.001[a] | 0.012±0.001[a] | n.d. | 0.064±0.008[a] | 0.001±0.001[a] | 0.329±0.005[a] |
| **NN2B** | 590.6±22.0[a] | 58.5±1.4[b] | 276.7±16.9[a] | 507.3±2.9[a] | 382.6±12.8[a] | 6.739±0.224[b] | 3.800±0.012[a] | 0.321±0.011[a] | 0.031±0.003[a] | 0.012±0.001[a] | 0.017±0.006[a] | 0.069±0.002[a] | 0.001±0.001[a] | 0.361±0.040[a] |
| **NN2C** | 520.2±3.6[b] | 60.4±0.2[a] | 272.4±15.1[a] | 494.1±9.3[a] | 374.8±1.5[a] | 6.313±0.362[b] | 3.507±0.099[a] | 0.291±0.033[a] | 0.031±0.001[a] | 0.015±0.002[a] | 0.035±0.012[a] | 0.072±0.007[a] | 0.001±0.001[a] | 0.308±0.023[a] |
| **DM2A** | 478.1±4.3[c] | 43.4±0.5[a] | 152.4±3.3[c] | 365.5±3.2[b] | 350.2±8.4[a] | 8.267±0.848[a] | 5.599±0.099[a] | 0.219±0.024[a] | 0.046±0.005[a] | 0.027±0.005[a] | 0.094±0.013[a] | 0.042±0.002[a] | 0.001±0.001[a] | 0.289±0.015[a] |
| **DM2B** | 521.6±7.4[a] | 43.1±0.3[a] | 206.7±3.5[a] | 458.9±12.6[a] | 342.3±6.0[a] | 8.542±0.123[a] | 3.509±0.108[b] | 0.224±0.012[a] | 0.042±0.001[a] | 0.028±0.003[a] | 0.142±0.061[a] | 0.045±0.001[a] | 0.001±0.001[a] | 0.282±0.014[a] |
| **DM2C** | 505.2±2.5[b] | 43.4±0.4[a] | 191.7±2.4[b] | 458.3±6.6[a] | 347.8±6.5[a] | 8.121±0.032[a] | 3.683±0.540[b] | 0.200±0.023[a] | 0.038±0.004[a] | 0.031±0.001[a] | 0.061±0.031[a] | 0.056±0.012[a] | 0.001±0.001[a] | 0.290±0.012[a] |
| **DA2A** | 519.7±15.4[a] | 31.2±0.5[b] | 208.2±8.1[a] | 470.8±29.1[a] | 248.9±7.5[a] | 8.298±0.034[a] | 3.619±0.057[b] | 0.270±0.027[a] | 0.029±0.004[a] | 0.027±0.011[a] | 0.037±0.003[a] | 0.026±0.003[a] | 0.001±0.001[a] | 0.291±0.010[a] |
| **DA2B** | 450.3±38.4[b] | 30.8±0.8[b] | 174.3±5.7[b] | 400.1±17.1[b] | 249.4±7.9[a] | 8.154±0.476[a] | 3.818±0.324[a] | 0.225±0.030[a] | 0.031±0.003[a] | 0.018±0.009[a] | 0.041±0.002[a] | 0.031±0.002[a] | 0.001±0.001[a] | 0.287±0.012[a] |
| **DA2C** | 493.1±12.5[a] | 33.6±0.1[a] | 151.7±1.0[c] | 380.8±11.3[c] | 254.6±9.1[a] | 6.361±2.360[b] | 3.923±0.403[a] | 0.277±0.053[a] | 0.029±0.001[a] | 0.030±0.002[a] | 0.040±0.002[a] | 0.026±0.004[a] | 0.001±0.001[a] | 0.281±0.002[a] |

Each sample was analyzed in quintuplicate and data are reported as means ± SD. Different superscript letters in the same column indicate significant differences between lots of each infant formula brands at a significance level of $p < 0.01$. IFs, infant formulas. n.d., not determined. The Se element in samples ME1C, NC1C, NN1C, NC2B and NN2A were not determined because their values were below the LOQ (LOQ = 0.015 $mg \cdot 100\ g^{-1}$).
