# Supplementary material for: Macrominerals and Trace Minerals in Commercial Infant Formulas Marketed in Brazil: Compliance With Established Minimum and Maximum Requirements, Label Statements, and Estimated Daily Intake
Source: Front Nutr. 2022 Apr 28;9:857698. doi: 10.3389/fnut.2022.857698 (PMC9096439; doi:10.3389/fnut.2022.857698)
Supplement: Supplementary file 7 [file Data_Sheet_7.PDF]

Supplementary file S7

Table S7. Estimated daily intake of the minerals present in phase 1 and phase 2 infant formulas and dietary reference intake values.

	**Macrominerals (mg·day^{-1})**					**Trace minerals (mg·day^{-1})**								
**AI**	**Ca**	**Mg**	**Na**	**K**	**P**	**Fe**	**Zn**	**Cu**	**Cr**	**Mo**	**Se**	**I**	**Co**	**Mn**
**(0-6 M)**	200	30	110	400	100	0.27	2	0.2	0.0002	0.002	0.015	0.11	ND	0.003
EDI of phase 1 infant formulas (mg·day^{-1})														
**ME1A**	338.3	55.7	152.1	522.5	355.4	6.379	5.178	0.551	0.030	0.032	0.029	0.049[ϰ]	0.001	0.313
**ME1B**	481.5	56.9	169.0	454.2	372.8	5.881	3.836	0.202	0.039	0.049	0.024	0.044[ϰ]	0.001	1.018
**ME1C**	437.7	51.4	183.2	422.2	314.5	6.086	2.877	0.169[Є]	0.031	0.045	ND	0.033[ϰ]	0.001	0.370
**NC1A**	282.9	69.0	125.2	381.9[δ]	222.7	7.045	5.019	0.450	0.034	0.012	0.045	0.074[ϰ]	0.001	0.411
**NC1B**	356.9	75.8	178.3	443.3	245.0	6.747	4.981	0.432	0.034	0.010	0.045	0.060[ϰ]	0.001	0.403
**NC1C**	286.7	66.7	133.5	394.8[δ]	219.1	6.581	4.984	0.431	0.025	0.012	ND	0.069[ϰ]	0.001	0.421
**NN1A**	396.2	73.9	209.7	430.7	297.7	6.051	5.094	0.436	0.032	0.011	0.043	0.079[ϰ]	0.001	0.453
**NN1B**	480.9	74.1	238.3	504.5	292.2	8.329	5.892	0.518	0.029	0.013	0.023	0.076[ϰ]	0.001	0.487
**NN1C**	469.8	71.2	228.6	490.4	311.9	6.321	5.175	0.428	0.029	0.014	ND	0.079[ϰ]	0.001	0.483
**DM1A**	409.6	42.2	165.7	370.4[δ]	270.1	7.217	3.411	0.349	0.027	0.027	0.039	0.045[ϰ]	0.001	0.353
**DM1B**	438.8	43.3	149.5	365.7[δ]	293.4	6.150	3.072	0.351	0.029	0.027	0.045	0.042[ϰ]	0.001	0.342
**DM1C**	438.1	45.4	161.0	377.3[δ]	297.1	7.458	3.391	0.343	0.024	0.028	0.051	0.039[ϰ]	0.001	0.337
**DA1A**	477.8	35.9	179.9	459.4	270.8	6.880	3.304	0.279	0.030	0.013	0.044	0.052[ϰ]	0.001	0.379
**DA1B**	436.5	36.3	159.0	399.1[δ]	256.9	7.173	3.486	0.314	0.026	0.014	0.048	0.048[ϰ]	0.001	0.360
**DA1C**	514.7	42.0	179.4	425.1	321.2	7.300	4.039	0.362	0.023	0.012	0.025	0.042[ϰ]	0.001	0.408
EDI of phase 2 infant formulas (mg·day-1)														
**AI/EAR[*]**	**Ca**	**Mg**	**Na**	**K**	**P**	**Fe**	**Zn**	**Cu**	**Cr**	**Mo**	**Se**	**I**	**Co**	**Mn**
**(7-12 M)**	**260**	**75**	**370**	**700**	**275**	**11/6.9[*]**	**3/2.5[*]**	**0.22**	**0.005**	**0.003**	**0.02**	**0.13**	**ND**	**0.6**
**ME2A**	817.7	79.4	321.2[α]	785.4	541.0	9.676	4.817	0.308	0.047	0.043	0.034	0.058[ж]	0.002	0.447[ҽ]
**ME2B**	791.6	67.2[π]	339.6[α]	795.8	459.3	9.373	3.066	0.228	0.050	0.044	0.046	0.066[ж]	0.002	0.440[ҽ]
**ME2C**	800.1	66.8[π]	323.7[α]	757.4	478.5	9.519	4.181	0.183[T]	0.046	0.042	0.049	0.071[ж]	0.002	0.411[ҽ]
**NC2A**	700.2	79.4	281.0[α]	615.9[ϒ]	453.3	8.975	4.414	0.403	0.042	0.006	0.055	0.061[ж]	0.002	0.348[ҽ]
**NC2B**	718.9	80.9	271.0[α]	637.1[ϒ]	463.7	9.805	4.826	0.427	0.043	0.014	ND	0.074[ж]	0.002	0.395[ҽ]
**NC2C**	733.1	82.2	283.8[α]	704.8	472.0	8.784	4.557	0.401	0.042	0.006	0.056	0.064[ж]	0.002	0.361[ҽ]
**NN2A**	556.1	58.5[π]	263.0[α]	494.7[ϒ]	380.3	7.108	3.756	0.329	0.031	0.012	ND	0.063[ж]	0.002	0.324[ҽ]
**NN2B**	583.0	57.7[π]	273.1[α]	500.7[ϒ]	377.7	6.651	3.750	0.317	0.030	0.012	0.020	0.069[ж]	0.001	0.356[ҽ]
**NN2C**	513.4	59.6[π]	268.9[α]	487.7[ϒ]	369.9	6.231	3.461	0.288	0.031	0.015	0.035	0.071[ж]	0.001	0.304[ҽ]
**DM2A**	612.0	55.5[π]	195.0[α]	467.8[ϒ]	448.3	10.582	7.167	0.280	0.060	0.035	0.120	0.054[ж]	0.002	0.370[ҽ]
**DM2B**	667.7	55.1[π]	264.6[α]	587.4[ϒ]	438.1	10.934	4.492	0.287	0.054	0.036	0.181	0.057[ж]	0.001	0.361[ҽ]
**DM2C**	646.6	55.0[π]	245.4[α]	586.6[ϒ]	445.2	10.395	4.714	0.257	0.049	0.039	0.078	0.072[ж]	0.002	0.371[ҽ]
**DA2A**	711.9	42.7[π]	285.2[α]	645.0[ϒ]	341.0	11.369	4.958	0.370	0.039	0.038	0.051	0.036[ж]	0.001	0.399[ҽ]

**DA2B**	617.0	41.3[π]	238.7[α]	548.1[ϒ]	341.7	11.171	5.230	0.308	0.042	0.024	0.056	0.042[ж]	0.001	0.393[ҽ]
**DA2C**	675.6	46.0[π]	207.8[α]	521.7[ϒ]	348.8	8.714	5.375	0.380	0.040	0.041	0.055	0.036[ж]	0.001	0.384[ҽ]

The EDI of each mineral content in infant formula was calculated by multiplying the value obtained from minerals (value shown in table S6 was divided by 100 to convert the concentration to mg/g) and the daily consumption recommended on the labels (Table S3). AI, Adequate Intake; EAR, Estimated Average Requirement; ND, Not Determinable. Values followed by symbol (π) indicates estimated daily intakes below AI/EAR (7-12 M) for Mg. Values followed by symbol (α) indicates estimated daily intakes below AI/EAR (7-12 M) for Na. The symbol (δ) indicates estimated daily intakes below AI (0-6 M) for K. The symbol (ϒ) indicates estimated daily intakes below AI/EAR (7-12 M) for K. Values followed by symbol (C) indicates estimated daily intakes below AI (0-6 M) for Cu. Values followed by symbol (T) indicates estimated daily intakes below AI/EAR (7-12 M) for Cu. The symbol (θ) indicates estimated daily intakes below AI (0-6 M) for Cr. The symbol (ω) indicates estimated daily intakes below AI/EAR (7-12 M) for Cr. The symbol (ϰ) indicates estimated daily intakes below AI (0-6 M) for I. The symbol (ж) indicates estimated daily intakes below AI/EAR (7-12 M) for I. The symbol (ҽ) indicates estimated daily intakes below AI/EAR (7-12 M) for Mn. *An EAR for Fe and Zn have been derived for this age group. Source: (38).
